# Lunacy Scandals

**Published:** 1900-06-16

**Authors:** 


### The Institutional Workshop.

### LUNACY SCANDALS.

Some of the daily papers comment on the unjustifiable system of "gratuities" which the St. Pancras Guardians have brought to light as existing between their relieving officers and medical men and the proprietors of private asylums.

Under the heading of "Wholesale Bribery in St. Pancras District," the following appears in the *Daily Mail*:—

"All the relieving officers in St. Pancras Parish were yesterday severely censured for having received bribes in connection with the certification and admission of lunatics.

"The censure was the result of a report presented to the Guardians by Mr. Fred Purchase, chairman of the Relief Committee.

"He said that out of the guinea fee received by doctors who certified cases of lunacy the relieving officers admitted having received 5s. from the medical officers of the board, and 1s. upwards from other medical men.

"Doctors who had refused to give 'presents' had been boycotted by the relieving officers.

"Nearly all the lunatics from St. Pancras had been sent to two private asylums—Hoxton House and Bethnal House—to whom the board paid over £12,000 a year. Those two houses had charged the board £2 2s. per patient. Other parishes were only charged 19s. 3d. per patient.

"St. Pancras had been paying more than four times as much as would have been the case had the patients been sent to the county asylum, where the charge was 9s. 11d. per patient.

"The relieving officers admitted having received sums varying from £2 to 10s. from the officials of those two asylums.

"'This system,' Mr. Purchase added, 'has been going on for 30 years. I do not care to say so myself, but the man in the street may well argue that lunacy cases have been manufactured. At any rate, it is remarkable that while St. Pancras has 1,600 lunacy cases, Islington, a larger parish, has only slightly over a thousand.'

"In severely censuring the officers the Chairman said their public career stood in danger of becoming a complete wreck. He hoped, for their sakes, that the Local Government Board would take as merciful a view of their conduct as the Guardians had taken."

We have not yet heard the defence of the medical officers nor of the private asylum proprietors; but it would seem that the relieving officers, or some of them, have admitted receiving money; and have been severely censured by the guardians. If the facts be granted, it seems to us that immediate dismissal of the officers should at once have taken place, and orders given to the new relieving officers that such medical officers as had been known to offer gratuities should never again be allowed to examine lunatics, and that no patients should be sent to the asylums named. Further, the giving of "tips" by medical men or by the proprietors of asylums, when these are medical men, might well form a subject of inquiry by the General Medical Council.

The innuendo that lunacy cases have been "manufactured" is one which we cannot for a moment believe; but private asylum proprietors who give money to the relieving officers can hardly wonder that they are suspected by people ignorant of the working of our asylum system. Neither do we believe that many medical men give these "bribes," and we are glad to find that one of St. Pancras Guardians stated that some medical men had been boycotted rather than give them.

To go a little deeper into the matter, it is impossible not to see that much blame rests on the London County Council for not providing sufficient asylum accommodation for their lunatics; the consequences of which neglect are that St. Pancras pays £2 2s. a week each for their lunatics sent to private asylums instead of 10s. in the County Asylum.—Ed. The Hospital.

